# Healthcare-Associated Infections in Subjects With Severe Acquired Brain Injury: The Effect of Microbial Colonization on the Functional Outcome. Data From a Multicenter Observational Study

**DOI:** 10.3389/fneur.2020.563275

**Published:** 2020-11-10

**Authors:** Michelangelo Bartolo, Chiara Zucchella, Hend Aabid, Beatrice Valoriani, Mauro Mancuso, Domenico Intiso

**Affiliations:** ^1^Neurorehabilitation Unit, Department of Rehabilitation, HABILITA Zingonia, Bergamo, Italy; ^2^Neurology Unit, University Hospital of Verona, Verona, Italy; ^3^Medicine Unit, Ospedali Riuniti della Valdichiana, Presidio di Nottola, Siena, Italy; ^4^Tuscany Rehabilitation Clinic, Arezzo, Italy; ^5^Physical and Rehabilitative Medicine Unit, NHS-USL Toscana Sud Est, Grosseto, Italy; ^6^Unit of Neurorehabilitation and Rehabilitation Medicine, Istituto di Ricovero e Cura a Carattere Scientifico “Casa Sollievo della Sofferenza, ” Foggia, Italy

**Keywords:** healthcare-associated infections (HAI), neurorehabilitation, severe acquired brain injury, antibiotic therapy, antibiotic resistance, microbial colonization, outcome, rehabilitation

## Abstract

**Background:** Hospital-acquired infections (HAIs) and microbial colonization are a worldwide serious threat for human health. Neurological patients with infections who undergo rehabilitation have a significantly poor recovery. The effect of microbial colonization on the functional outcome in severe acquired brain injury (sABI) subjects is still unclear.

**Aim:** The aim of this multicenter observational study was to describe the clinical impact of HAIs and colonization on the functional outcome of sABI subjects admitted to inpatient neurorehabilitation.

**Methods:** Patients were assigned to three groups: infected (INF), not infected (noINF), and colonized (COL). The Glasgow Coma Scale (GCS), the Rancho Los Amigos Levels of Cognitive Functioning Scale, Disability Rating Scale, and modified Barthel Index (mBI) assessments were performed both at admission and discharge.

**Results:** Two hundred sixty-five (92 female/173 male) patients were enrolled: 134 were assigned to INF, 63 to COL, and 68 to noINF. In the INF group, 231 culture specimens were found positive for bloodstream (44.2%), respiratory tract (25.5%), urinary tract (18.6%), gastrointestinal tract (8.3%), skin (3%), and cerebrospinal fluid (0.4%) infections. After rehabilitation, all groups showed a significant improvement in all assessment tests, except for the noINF group that did not show any improvement in GCS. Both noINF and COL groups showed a significantly higher gain in mBI than the INF group (*p* = 0.000). The COL group showed a significantly higher gain than the noINF group in GCS (*p* = 0.001). A significantly lower improvement was detected in the INF group than the COL and noINF groups. The rate of patients who needed functional isolation was higher in the INF group than the COL group. Length of stay (LOS) (in days) was 56 ± 50.7, 88.3 ± 55, and 101.3 ± 73.6 for noINF, INF, and COL groups, respectively. The number of deaths in the INF group was significantly higher (24.6%) than the noINF group (7.4%) (*p* = 0.005) and comparable to the COL group (19%).

**Conclusion:** Colonized sABI patients obtained a similar functional outcome to that of subjects who had no infections, even if they needed a significantly higher LOS.

## Introduction

Hospital-acquired infections (HAIs) are a serious health problem (1). Each year, there are thousands of cases of infections in hospitals and care facilities, where there are obviously more vulnerable subjects (premature, immunosuppressed, elderly) who are more susceptible to develop infections caused by instrumental maneuvers and the placement of a number of devices, such as cannulas and catheters (2–4).

Among the factors that worsen this condition and hinder recovery, colonization of microorganisms resistant to antibiotics, also called “multidrug resistant” (MDR), has a prominent role as they are resistant to one or more classes of antimicrobial agents. The term “colonization” defines the presence of microorganisms in or on a host that grow and multiply but without any clinical evidence of infection (without tissue invasion or immune response) (5, 6). Colonization is a serious threat given its impact on morbidity and mortality and its potential for dissemination of microbial resistance (7).

Data on colonization are available for intensive care units (ICUs) (8–11), care settings (12), or tertiary hospitals (13, 14), while information about rehabilitation settings—in particular neurorehabilitation—is very limited or lacking. In this regard, a recent study investigating the frequency of intestinal colonization by *Clostridium difficile* or extended-spectrum beta-lactamase–producing Enterobacteriaceae agents in some rehabilitation clinics in Germany showed that colonization rates for these pathogens were higher in neurorehabilitation wards than in other rehabilitation clinics (15). In Italy, a single-center study reported that about 10.2% of patients admitted to that neurorehabilitation unit became colonized by carbapenemase-producing Enterobacteriaceae (CPE) organisms (16). These findings were further confirmed by another study that described the burden of CPE in an Italian neurorehabilitation institute during a 6-year period, showing that overall 9.3% of patients were CPE rectal carriers at admission, and 8.1% became colonized during hospitalization (17).

Patients admitted to neurorehabilitation units, particularly those suffering from severe acquired brain injury (sABI), are more at risk of developing infections and microbial colonization. The main causes are the higher intensity of care and the presence of predisposing factors including greater age, multiple comorbidities, poor functional status, previously repeated and/or prolonged courses of antibiotic exposure, prolonged stay and/or frequent readmission to acute care facilities, and use of invasive medical devices or mechanical ventilation (18–21). Moreover, the brain injury–induced immunosuppression syndrome, which manifests as a consequence of dysregulation of the brain–immune interactions, may facilitate infections and colonization (22–24). Consistently, a surveillance program implemented in a large Italian long-term acute care rehabilitation facility revealed that sABI patients were at much higher risk of developing CPE bacteremia compared to other subpopulations admitted to rehabilitation settings. Indeed, sABI patients can show a rate of *Klebsiella pneumoniae* carbapenemase-producing infection up to 90% (25). The detrimental impact of HAIs on the rehabilitative outcome of sABI patients and their economic burden have been extensively described (26–28). Likewise, colonization has been analyzed in acute care departments, including ICU and surgical departments, but it has been poorly investigated in rehabilitation facilities, particularly in settings dedicated to rehabilitation of sABI patients. Moreover, as both epidemiological data and the rate at which colonized patients can spread pathogens into the environment have yet to be completely clarified, it is reasonable to suppose that the global impact of colonization on rehabilitative goals may be much greater than has been recognized so far.

Rollnik et al. (29) reported that functional recovery of neurological patients who underwent early rehabilitation and were colonized by methicillin-resistant *Staphylococcus aureus* (MRSA) was worse than MRSA-negative patients. Recently, a European survey involving 45 hospitals and aiming to assess the management and the outcome of colonized patients in rehabilitation facilities reported that the estimated MDR colonization prevalence rate is of 31% and that there is a widespread use of functional isolation to manage these hospitalized patients (69%). Moreover, it was found that MDR-colonized patients usually waited longer for admission (36%) and showed a worse outcome (30).

The aim of the present study was to evaluate the frequency and the effect of HAIs and microbial colonization on the functional outcome of patients with sABI admitted to a dedicated rehabilitation facility.

## Materials and Methods

### Participants

All sABI patients consecutively admitted to three intensive neurorehabilitation centers from 1 September 1, 2018, to February 28, 2019, were enrolled in the study. sABI was defined as a central nervous system damage due to acute traumatic or non-traumatic (vascular, anoxic, neoplastic, or infectious) causes that led to a variably prolonged state of coma [Glasgow Coma Scale (GCS) ≤ 8] and produced a potentially wide range of impairments affecting the physical, cognitive, and/or psychological functioning (31–33). Exclusion criteria were as follows: previous neurological impairment; infected surgical wounds and/or infected pressure sores at admission; and positive infection indexes [increase of leukocytes number, erythrocyte sedimentation rate (ESR), C-reactive protein (CRP), procalcitonin] at admission to the rehabilitation unit. Subjects with encephalitis were enrolled when the infective process was considered clinically solved, and laboratory infection parameters were negative at admission.

All enrolled patients were divided into three different groups: infected (INF), colonized (COL), and non-infected group (noINF). The study waived the written informed consent because it was a retrospective data analysis, relying on measurements and data acquisition applied as part of routine care and derived from medical charts. The study was notified to the local ethics committee.

### Study Design and Procedures

This observational study was conducted as a part of the standard clinical practice. Patients' information was entered into the study database anonymously.

Clinical and functional data were derived from patients' charts and collected in a computer database that included the following variables: age, sex, etiology of the brain damage, site of lesion, comorbidities, presence of central venous catheter (CVC), percutaneous endoscopic gastrostomy (PEG), urinary catheter, tracheostomy, and pressure sores. Moreover, surgical or invasive procedures performed during acute care were recorded including ventriculoperitoneal shunt, craniotomy, and cranioplasty. Data about HAIs were recorded including frequency, species of microorganisms, types of culture specimens (hematic, urinary, bronchial and/or tracheostomy secretions, cutaneous, feces, cerebrospinal fluid, CVC), and antimicrobial therapy (drugs, days of duration).

For the purpose of this study, patients were defined as follows:

- Infected (INF group): when the patients' body tissues became invaded by microorganisms resulting in disease, according to the Centers for Disease Control and Prevention/National Healthcare Safety Network surveillance definitions (1), together with the presence of positive specimens and clinical signs (increase in the number of leukocytes, ESR, CRP, procalcitonin). In the presence of pneumonia also radiological evidence (such as new or progressive and persistent infiltrates, consolidation, or cavitation) was considered. If patients showed more than one type of pathogens, they were categorized as infected due to multiple pathogens;
- Colonized (COL group): despite the presence of cultures positive for pathogens, the agents did not cause a specific immune response or infection in the host, and no antibiotic therapy was needed (34, 35). All enrolled patients were identified as “colonized” at admission, according to the indications by the ICU and acute wards, where they were assessed before admission to a dedicated intensive rehabilitation unit. Confirmation of the colonization status was linked to the presence of positive pathogenic cultures (performed again in neurorehabilitation) in the absence of clinical signs (leukocytosis, ESR, PCR, fever);
- Non-infected (noINF group) when the previous conditions were not satisfied, and no signs of infection were detected at admission or during stay in the neurorehabilitation ward.

To evaluate patients' clinical and functional status, the following tests were administered at admission (T0) and discharge (T1): GCS (36), the Rancho Los Amigos Levels of Cognitive Functioning Scale (LCF) (37), Disability Rating Scale (DRS) (38), modified Barthel Index (mBI) (39).

Data about functional isolation, suspension of rehabilitation treatment (number of sessions), hospital length of stay (LOS), and mortality were also collected.

### Statistical Analysis

Demographic and clinical variables were summarized by means of descriptive statistics.

The assumption of normal distribution for each continuous variable was checked by means of the Shapiro–Wilk test. As the data deviated from the normal distribution, the analysis was performed by means of nonparametric statistics, and the distribution was described as median values and the 25th and 75th quartiles.

Categorical variables were reported as frequencies and percentages and compared using the χ^2^ test or the Fisher exact test; when statistical significant differences emerged between the three groups, the *post hoc* analysis with the χ^2^ was performed.

With regard to clinical and functional scales, changes within groups [admission (T0)—discharge (T1)] were investigated using the Wilcoxon signed rank test. Intergroup comparisons were performed by means of the Kruskal–Wallis test, using the Mann–Whitney *U*-test to make *post hoc* comparisons; for *post hoc* analysis, the Bonferroni correction was applied. For all the clinical scales, besides admission and discharge values, also the “gain” parameter [difference between discharge (T1) and admission (T0) score] for each group was calculated and compared.

All tests were 2-sided, and the level of significance was set at 0.05 for intragroup comparison, whereas for intergroup comparison with *post hoc* analysis, the significance was set at 0.017 (according to Bonferroni correction).

Data processing and statistical analyses were performed with SPSS Statistics for Windows (version 18.0).

## Results

Among 288 consecutive sABI patients admitted to the three neurorehabilitation centers, 23 (8 female, 15 male) patients were excluded because of previous neurological impairments (16), infected wounds (1) or pressure sores (3), and positive infection indexes (3); 265 (92 female, 173 male) patients with a mean age of 63.6 ± 16.1 years were enrolled in the study. Of these, 134 patients (50.6%) were included in the INF group, 63 (23.8%) in the COL group and 68 (25.6%) in the noINF group. Patients' demographic and clinical features are shown in Table 1. The intergroup comparison showed the following significant differences: the percentage of patients coming from the neurosurgery unit was higher in the COL group than in the INF group (*p* = 0.001); the noINF group showed a higher rate of traumatic etiology than the INF group (*p* = 0.004). At admission, INF and COL patients showed worse clinical conditions than noINF patients. Indeed, devices were significantly more frequent in the INF group and the COL group than in the noINF group, in particular CVC (*p* = 0.014) and PEG and/or CV presence (*p* = 0.012 and *p* = 0.008, respectively). No statistical significance was found for any other intergroup comparison about demographic and clinical features.

**Table 1:** Patients' demographic and clinical features.

		**INF group (*n* = 134)**	**COL group (*n* = 63)**	**NoINF group (*n* = 68)**
Age, years [25th; 75th quartiles]		68 [59; 76]	66 [52; 75]	60.5 [52.25; 74]
Gender, *n* (%)	Female	43 (32.1)	27 (42.9)	22 (32.4)
	Male	91 (67.9)	36 (57.1)	46 (67.6)
Time from acute event, days [25th; 75th quartiles]		26 [20.75; 46.50]	31 [20; 53]	27.5 [19; 46.75]
LOS in neurorehabilitation, days [25th; 75th quartiles]		73 [44;127]^b^	79 [43; 180]^c^	40 [22.75; 70.5]
Discharge wards, *n* (%)	Intensive care units	75 (55.9)	34 (53.9)	33 (48.5)
	Neurosurgery	14 (10.4)^a^	19 (30.1)	10 (14.7)
	Neurology	16 (11.9)	4 (6.3)	8 (11.7)
	Stroke unit	13 (9.7)	5 (7.9)	7 (10.2)
	Surgery	2 (1.4)	0	2 (2.9)
	Medicine	14 (10.4)	1 (1.5)	8 (11.7)
Comorbidities, *n* (%)	Cardiovascular	52 (38.8)	17 (27)	21 (30.9)
	Dysmetabolic/endocrine	7 (5.2)	1 (1.6)	3 (4.4)
	Neoplastic	2 (1.5)	1 (1.6)	0
	Psychiatric	3 (2.2)	0	0
	Neurologic	6 (4.5)	0	3 (4.4)
	Multiple	29 (21.6)	22 (34.9)	14 (20.6)
	Other	7 (5.2)	2 (3.2)	2 (2.9)
Etiology, *n* (%)	Ischemic	30 (22.4)	10 (15.9)	13 (19.1)
	Hemorrhagic	48 (35.8)	21 (33.3)	15 (22)
	Infective	7 (5.2)	2 (3.2)	1 (1.5)
	Traumatic	25 (18.7)^b^	19 (30.2)	26 (38.2)
	Hypoxic	23 (17.2)	7 (11.1)	12 (17.7)
	Neoplastic	1 (0.7)	4 (6.3)	1 (1.5)
Lesion site, *n* (%)	Diffuse damage	31 (23.2)	10 (15.9)	18 (26.5)
	Right hemisphere	26 (19.4)	15 (23.8)	17 (25)
	Left hemisphere	33 (24.6)	15 (23.8)	12 (17.6)
	Bilateral	15 (11.2)	6 (9.5)	10 (14.7)
	Posterior cranial fossa	4 (3)	1 (1.6)	0
	Brainstem	1 (0.7)	5 (7.9)	1 (1.5)
	Basal ganglia	6 (4.5)	8 (12.7)^a, c^	2 (2.9)
	Multiple sites	16 (11.9)	2 (3.2)	8 (11.8)
	Other	2 (1.5)	1 (1.6)	0
Devices, *n* (%)	Central venous catheter	61 (45.5)^b^	21 (33.3)	18 (26.5)
	Percutaneous endoscopic gastrostomy	49 (36.6)	35 (55.6)^c^	22 (32.4)
	Urinary catheter	127 (94.8)	63 (100)	59 (86.8)
	Tracheostomy	104 (77.6)	46 (73)	47 (69.1)
	Ventriculoperitoneal shunt	5 (3.7)	7 (11.1)^c^	1 (1.5)

*The 25th and the 75th quartiles are given in square brackets*.

Significance p < 0.017:

a *INF vs. COL*,

b *INF vs. noINF*,

c *COL vs. noINF*.

*Where not otherwise indicated, the intergroup comparison must be considered as “not statistically significant”*.

Among patients discharged from ICU, 30 (21.1%) needed ventilator care during ICU stay: 17 (22.6%) in the INF, 6 (18.1%) in the noINF, and 7 (20.6%) in the COL group; no statistical differences among groups were found. During ICU stay, 87 (61.2%) patients took antibiotic therapies: 51 (68%) in the INF, 4 (12.1%) in the noINF, and 32 (94.1%) in the COL group, with the following statistical significant differences: INF vs. noINF, *p* = 0.000; INF vs. COL, *p* = 0.006; noINF vs. COL, *p* = 0.000.

In the INF group, 231 positive culture specimens were found: 95 (41.2%) blood, 42 (18.2%) urine, 35 (15.2%) oropharyngeal swabs, 19 (8.2%) feces, 15 (6.5%) tracheostomy, 9 (3.9%) expectorated sputum, 7 (3%) CVC, 7 (3%) skin, 1 (0.4%) cerebrospinal fluid, and 1 (0.4%) vaginal swab. Furthermore, the following infections were identified: 102 bloodstream infections (44.2%) [27 (11.6%) catheter-related]; 59 respiratory tract infections (25.5%) [pneumonia 35 (15.2%), upper airways 24 (10.3%)]; 43 urinary tract infections (18.6%); 19 gastrointestinal infections (8.3%); 7 skin infections; and 1 cerebrospinal fluid infection (0.4%).

Single isolated pathogens were as follows: *K. pneumoniae* (11.2%), *Pseudomonas aeruginosa* (6%), *Acinetobacter baumannii* (4.5%), *C. difficile* (4.5%), *Enterococcus* (3%), *Staphylococcus* (3%), *Escherichia coli* (3%), *Proteus mirabilis* (2.2%), *Mycobacteria* (0.7%), *Morganella morganii* (0.7%), *Providencia* (0.7%), *Serratia* (0.7%), and *Enterobacter cloacae* (0.7%). Seventy-four patients (55.2%) had multiple microorganisms, while in five cases (3.7%), no pathogens were identified. Fifty (37.3%) patients presented recurrent infections during their stay in neurorehabilitation ward. Patients in the INF group were administered antibiotic therapy on average for 20.1 ± 10.9 days.

In the COL group, 77 culture specimens were taken, and the isolated bacteria were *E. coli* (9.5%), *K. pneumoniae* (9.5%), *P. aeruginosa* (6.3%), *P. mirabilis* (3.2%), *A. baumannii* (3.2%), and *M. morganii* (3.2%). Thirty-three (52.4%) specimens revealed multiple microorganisms; in six (9.5%) cases, no pathogens were identified, and two missing data were recorded.

### Functional Outcome

At admission, INF patients' scores were significantly lower than those of noINF patients in all clinical scales (GCS, LCF, DRS, mBI). Likewise, significantly lower scores than the COL group (*p* < 0.005) were detected in all measurements, except for the LCF score. Clinical scale scores of the COL group were comparable to those of the noINF group, except for the DRS value that was significantly lower in the COL group (*p* = 0.001; Table 2). After rehabilitation, all groups showed a significant improvement in all assessment measures, except for the noINF group that did not show any significant improvement in GCS. However, the improvement of INF patients was significantly lower than that of COL and noINF patients at discharge (all *p* < 0.009), while no significant differences between the COL group and noINF group were observed (Table 2).

**Table 2:** Outcome measures: between-group analysis.

**Outcome measures**		**INF group**	**COL group**	**NoINF group**
**GCS**	Admission	8 [6; 11]^a^^,^ ^b^	11 [8; 14]	13 [8; 15]
	Discharge	12 [8; 15]^a^^,^ ^b^	14 [12; 15	15 [11; 15]
	Gain	1 [0; 4]	1 [0; 4]^c^	0 [0; 2]
**LCF**	Admission	3 [2; 4]^b^	3 [2; 5]	4 [3; 6]
	Discharge	4 [2; 6]^a^^,^ ^b^	5 [3.75; 8]	6 [4; 7]
	Gain	1 [0; 2]	1 [0; 2]	1 [0; 2]
**DRS**	Admission	20 [9; 26]^a^^,^ ^b^	8 [7; 9]^c^	14 [7; 20]
	Discharge	12 [8; 20]^a^^,^ ^b^	6 [5; 8]	6 [3; 10]
	Gain	−1 [−6; 0]^b^	−2 [−4; 0]^c^	−4 [−11; −1]
**mBI**	Admission	0 [0; 0]^a^^,^ ^b^	0 [0; 2]	0 [0; 17.75]
	Discharge	0 [0; 11.25]^a^^,^ ^b^	25 [0; 75]	35 [4; 82]
	Gain	0 [0; 10]^a^^,^ ^b^	15 [0; 56]	15 [0; 60]

*The 25th and the 75th quartiles are given in square brackets*.

Significance p < 0.017:

a *INF vs. COL*,

b *INF vs. noINF*,

c *COL vs. noINF*.

*Where not otherwise indicated, the intergroup comparison must be considered as “not statistically significant”*.

Comparing the COL group and the noINF group, the COL group showed a significantly higher gain than the noINF group in GCS (*p* = 0.001). Both the noINF and the COL groups showed a significantly higher gain in mBI than the INF group (*p* = 0.000). Furthermore, the noINF group showed a significantly higher gain in DRS than the INF group (*p* = 0.009) and the COL group (*p* = 0.011; Table 2). NoINF patients did not miss any rehabilitation session, while the average number of skipped rehabilitation sessions was 0.49 ± 1.3 for the INF group and 0.67 ±1.3 for COL group. However, no significant differences among the three groups were observed.

The rate of patients who needed functional isolation was significantly higher in the INF group (68; 50.7%) than in the COL group (19; 30.2%) (*p* = 0.01).

Although the INF group showed a higher death rate (33; 24.6%) than the COL group (12; 19%), no statistical differences were observed. Likewise, no difference was detected between the COL group and the noINF group (5; 7.4%) (*p* = 0.005). On the other hand, a significantly higher death rate was observed in the INF group than the noINF group.

## Discussion

Colonized sABI patients were frequent in our dedicated rehabilitation unit, and their functional outcome was similar to that of subjects without infections, even if these patients needed a significantly higher LOS. On the other hand, the INF group showed a significantly lower improvement and a higher LOS and rate of mortality than noINF subjects.

It is well-known that neurological patients with infections undergoing rehabilitation have a significantly poor recovery. In this respect, several studies have demonstrated that HAIs are an independent predictor of poor functional outcome and mortality; moreover, it may delay hospital discharge and cause an increase in the costs of care and the use of medical resources (40, 41). This study confirms that INF patients have a significantly poorer outcome and a higher LOS and mortality rate than noINF subjects. Notwithstanding, INF patients obtained a significantly functional improvement by intensive rehabilitation interventions. As expected, the INF group performed the highest number of culture specimens, which revealed bloodstream, respiratory tract, and urinary tract infections. Furthermore, a third of the patients of this group developed multiple concurrent infections during their rehabilitation stay.

Although HAIs and microbial colonization are the most relevant public health problems particularly in the ICU setting, this phenomenon is progressively extending to the neurorehabilitation setting and may represent a troublesome complication in sABI patients (21). Some information on the role and effect of colonization on the rehabilitation process and the outcome of these patients is available. A retrospective study analyzing medical records of neurological patients who underwent early rehabilitation in a large rehabilitation facility in Northern Germany revealed that the functional outcome of MRSA-positive patients was worse than that of MRSA-negative patients (29). These data were confirmed by the authors of a multicenter study that enrolled 754 early neurological patients (30). By contrast, the present study shows that there are no significant differences in functional improvement between COL patients and noINF patients, after rehabilitation. These conflicting results may be due to the cohorts of enrolled patients and type of agents responsible for colonization. Indeed, the colonized subjects in the study by Rollnik et al. (30) had a poor functional status and higher morbidity at admission, while no differences in clinical and functional status were observed in our patients. On the other hand, the frequency of colonized subjects was quite similar to the one detected by Rollnik et al. (30), but higher than that reported by two Italian studies that found 10.2% (16) and 9.3% (17) of microbial colonization in patients admitted to neurorehabilitation units. The reason for the lower frequency detected in these investigations might be the methodology and the aim of the studies, because only subjects colonized by CPE agents were investigated, whereas we collected all MDR organisms.

The present study shows that about a quarter of sABI patients admitted to our intensive neurorehabilitation units were colonized by MDR. This finding confirms that microbial colonization and HAIs represent worrying conditions in a dedicated rehabilitation setting, and it therefore requires attention and proper interventions.

LOS for COL patients, although not statistically different from that of INF patients, was the longest. In our opinion, this finding is probably related to the complex clinical pictures that COL patients usually show, as if the presence of pathogens, although not expressed in the form of symptoms, could determine a sort of “depletion” of resources (metabolic, functional), which makes recovery times longer.

Consistently with literature data, the use of devices (CVC, PEG, CV) was significantly greater in the COL and the INF groups than the noINF group: it is well-known that the use of invasive devices may be considerable in ICU patients, representing a serious threat to patient safety as devices may become vehicles of bacterial infections (42, 43). Moreover, it should be noted that in our sample a high rate of COL patients was discharged from the neurosurgery unit. The reason for this finding may be the greater use of antibiotic therapies with prophylactic purposes in these units to prevent postoperative infections. Although beneficial, this practice is controversial as preventive antibiotic therapy may favor the promotion of antibiotic-resistant strains of bacteria (44, 45). On the other hand, also COL patients discharged from ICU reported high percentages of antibiotic use during ICU stay, confirming literature evidences about the strict relationship between antibiotic use and microbial resistance.

A worrying factor that emerges from the present study is the rate of functional isolation use, which is significantly higher in the INF group than in the COL group. Our opinion is that the low rate of functional isolation in the COL group may be due to the fact that not all COL subjects were positive for those microorganisms that require isolation (i.e., *K. pneumoniae, A. baumannii*). However, other causing factors, such as organizational difficulties, lack of proper structure, spatial limitation, and level of education of healthcare personnel and the patients' relatives, cannot be excluded.

In this regard, despite frequent colonization, it has been previously recognized that care facilities including rehabilitation wards often do not have the same infection control resources as acute care units, nor is there a consensus on how colonized patients should be managed during rehabilitation stay (46). This aspect is particularly important in rehabilitation facilities, where the sharing of common spaces (e.g., gyms or areas for group therapy) and the close interaction with healthcare operators are basic elements of rehabilitation, but at the same time, they can facilitate the propagation of infections. On the other hand, previous studies have shown that in subjects who undergo rehabilitation, organizational measures such as functional isolation, besides several rising questions relating to the need for dedicated settings and proper care with related costs, may cause psychological distress symptoms including depression and anxiety in patients and their relatives (47, 48). Furthermore, because the rate of pathogen concentration was believed to be lower in colonized subjects, until not long ago the attention in preventing pathogen dissemination was mainly related to infected patients, and only recently, there has been a growing interest to better understanding the role of colonized patients (5, 35).

Although data from our study show that for both INF and COL subjects the number of missed rehabilitation sessions was negligible (on average, not even one per person), in order to reduce to a minimum the absence rate of patients from rehabilitation sessions in dedicated gyms, we suggest the use of protective equipment for both colonized patients and healthcare operators, the scheduling of activities at the end of the therapeutic sessions, and the adoption of appropriate cleaning procedures.

A limitation of the study is that we did not differentiate enrolled colonized patients on the basis of the effect that the MDR organisms had on the outcome and did not perform a regular rectal swab test to evaluate the potential diffusion of the colonization and transmission of resistant bacteria. On the other hand, in our opinion, also considering that the prevalence of sABI is not so high, the large cohort size, and the multicenter observational design, which is able to depict a real scenario, are two strengths of this study. In addition, this study draws attention to prevention, which is crucial to limit the spread of antibiotic-resistant bacteria among admitted patients. Colonization is becoming a major economic and health-related issue in all areas of healthcare, including neurological rehabilitation, as well as infections represent a big issue because their management determines complex clinical and organization approaches (patients isolation, pharmacological expenditure, individual protection devices, and other) with costs increasing for rehabilitation wards. On these bases, we believe that an evidence-based and multidisciplinary approach might be the solution. Specifically, in accordance with the World Health Organization (WHO) recommendations (49), we recommend some common and shared indications on how to manage colonization specifically in rehabilitation facilities: (1) prudential use of antibiotics, (2) implementation of surveillance systems, (3) changes to the infrastructure of rehabilitation facilities and to organizational models, (4) extensive use of protective equipment, and (5) promoting proper education and training in the management of colonized patients.

In conclusion, in our study, HAIs and microbial colonization were frequent in sABI patients admitted to our neurorehabilitation units. Colonized sABI patients had similar functional outcome to subjects without infections. Nevertheless, they showed an increased mortality rate and a significantly higher LOS than subjects without infections. Future studies should be aimed at investigating how rehabilitation settings should be organized to prevent the dissemination of colonization and HAIs.

## Data Availability Statement

The raw data supporting the conclusions of this article will be made available by the authors, without undue reservation.

## Ethics Statement

Ethical review and approval was required for the study on human participants in accordance with the local legislation and institutional requirements. Written informed consent for participation was not required for this study because it was based on previously collected data.

## Author Contributions

MB, CZ, and DI conceived the study, analyzed data, wrote, and revised the paper. MM conceived the study, wrote, and revised the paper. HA and BV collected data and revised the paper. All authors contributed to the article and approved the final version of the manuscript.

## Conflict of Interest

The authors declare that the research was conducted in the absence of any commercial or financial relationships that could be construed as a potential conflict of interest.

## References

[1] Horan TC Andrus M Dudeck MA CDC/NHSN surveillance definition of health care-associated infection and criteria for specific types of infections in the acute care setting. Am J Infect Control. (2008) 36:309–32. 10.1016/j.ajic.2008.03.002 18538699

[2] Allegranzi B Bagheri Nejad S Combescure C Graafmans W Attar H Donaldson L. Burden of endemic health-care-associated infection in developing coun-tries: systematic review and meta-analysis. Lancet. (2011) 377:228–41. 10.1016/S0140-6736(10)61458-4 21146207

[3] World Health Organization Report on the Burden of Endemic Health Care-Associated Infection Worldwide. (2011). Available online at: https://apps.who.int/iris/bitstream/handle/10665/80135/9789241501507_eng.pdf (accessed May 06, 2020).

[4] Cassini A Plachouras D Eckmanns T Sin MA Blank HP Ducomble T. Burden of six healthcare-associated infections on European population health: estimating incidence-based disability adjusted life years through a population prevalence-based modelling study. PLoS Med. (2016) 13:e1002150. 10.1371/journal.pmed.1002150 27755545 PMC5068791

[5] Casadevall A Pirofski LA. Host-pathogen interactions: basic concepts of microbial commensalism, colonization, infection and disease. Infect Immun. (2000) 68:6511–8. 10.1128/IAI.68.12.6511-6518.2000 11083759 PMC97744

[6] Public Health Agency of Canada (PHAC) Routine Practices and Additional Precautions for Preventing the Transmission of Infection in Healthcare Settings. Ottawa, ON: Her Majesty the Queen in Right of Canada (2013). Available online at: http://publications.gc.ca/collections/collection_2013/aspc-phac/HP40-83-2013-eng.pdf (accessed May 06, 2020).

[7] Cosgrove SE. The relationship between antimicrobial resistance and patient outcomes: mortality, length of hospital stay, and health care costs. Clin Infect Dis. (2006) 42 (Suppl. 2):S82–9. 10.1086/499406 16355321

[8] Jung JY Park MS Kim SE Park BH Son JY Kim EY. Risk factors for multidrug resistant Acinetobacter baumannii bacteremia in patients with colonization in the intensive care unit. BMC Infect Dis. (2010) 10:228. 10.1186/1471-2334-10-228 20670453 PMC2921386

[9] Buhl M Peter S Willmann M. Prevalence and risk factors associated with colonization and infection of extensively drug-resistant Pseudomonas aeruginosa: a systematic review. Expert Rev Anti Infect Ther. (2015) 13:1159–70. 10.1586/14787210.2015.1064310 26153817

[10] Detsis M Karanika S Mylonakis E. ICU acquisition rate, risk factors, and clinical significance of digestive tract colonization with extended-spectrum beta-lactamase-producing enterobacteriaceae: a systematic review and meta-analysis. Crit Care Med. (2017) 45:705–14. 10.1097/CCM.0000000000002253 28157141

[11] Hoang S Georget A Asselineau J Venier AG Leroyer C Rogues AM. Risk factors for colonization and infection by *Pseudomonas aeruginosa*in patients hospitalized in intensive care units in France. PLoS One. (2018) 13:e0193300. 10.1371/journal.pone.0193300 29522559 PMC5844558

[12] Xue Y Gyi AA. Predictive risk factors for methicillin-resistant *Staphylococcus aureus* (MRSA) colonisation among adults in acute care settings: a systematic review. JBI Libr Syst Rev. (2012) 10:3487–560. 10.11124/jbisrir-2012-16 27820009

[13] Marchaim D Chopra T Bogan C Bheemreddy S Sengstock D Jagarlamudi R. The burden of multidrug-resistant organisms on tertiary hospitals posed by patients with recent stays in long-term acute care facilities. Am J Infect Control. (2012) 40:760–5. 10.1016/j.ajic.2011.09.011 22285709

[14] Na'amnih W Adler A Miller-Roll T Cohen D Carmeli Y. Risk factors for recurrent *Clostridium* difficile infection in a tertiary hospital in Israel. Eur J Clin Microbiol Infect Dis. (2018) 37:1281–8. 10.1007/s10096-018-3247-1 29627951

[15] Arvand M Ruscher C Bettge-Weller G Goltz M Pfeifer Y. Prevalence and risk factors for colonization by *Clostridium* difficile and extended-spectrum β-lactamase-producing *Enterobacteriaceae* in rehabilitation clinics in Germany. J Hosp Infect. (2018) 98:14–20. 10.1016/j.jhin.2017.07.004 28705583

[16] Rossini A Di Santo SG Libori MF Tiracchia V Balice MP Salvia A. Risk factors for carbapenemase-producing *Enterobacteriaceae* colonization of asymptomatic carriers on admission to an Italian rehabilitation hospital. J Hosp Infect. (2016) 92:78–81. 10.1016/j.jhin.2015.10.012 26615459

[17] Tedeschi S Trapani F Liverani A Tumietto F Cristini F Pignanelli S The burden of colonization and infection by carbapenemase-producing *Enterobacteriaceae* in the neuro-rehabilitation setting: a prospective six-year experience. Infect Control Hosp Epidemiol. (2019) 40:368–71. 10.1017/ice.2018.344 30767830

[18] Lin MY Lyles-Banks RD Lolans K Hines DW Spear JB Petrak R. The importance of long-term acute care hospitals in the regional epidemiology of *Klebsiella pneumoniae* carbapenemase-producing *Enterobacteriaceae*. Clin Infect Dis. (2013) 57:1246–52. 10.1093/cid/cit500 23946222 PMC8383150

[19] Han JH Goldstein EJ Wise J Bilker WB Tolomeo P Lautenbach E Epidemiology of carbapenem-resistant *Klebsiella pneumoniae* in a network of long-term acute care hospitals. Clin Infect Dis. (2017) 64:839–44. 10.1093/cid/ciw856 28013258 PMC5399931

[20] Intiso D Fontana A Maruzzi G Tolfa M Copetti M Di Rienzo F. Readmission to the acute care unit and functional outcomes in patients with severe brain injury during rehabilitation. Eur J Phys Rehabil Med. (2017) 53:268–76. 10.23736/S1973-9087.16.04288-X 27585056

[21] Scarponi F Zampolini M Zucchella C Bargellesi S Fassio C Pistoia F. C.I.R.C.LE (Comorbidità in ingresso in riabilitazione nei pazienti con grave CerebroLEsione acquisita) study group. Identifying clinical complexity in patients affected by severe acquired brain injury in neurorehabilitation: a cross sectional survey. Eur J Phys Rehabil Med. (2019) 55:191–8. 10.23736/S1973-9087.18.05342-X 30543265

[22] Chamorro A Urra X Planas AM. Infection after acute ischemic stroke: a manifestation of braininduced immunodepression. Stroke. (2007) 38:1097–103. 10.1161/01.STR.0000258346.68966.9d 17255542

[23] Catania A Lonati C Sordi A Gatti S. Detrimental consequences of brain injury on peripheral cells. Brain Behav Immun. (2009) 23:877–84. 10.1016/j.bbi.2009.04.006 19394418

[24] Busl KM Nosocomial infections in the neurointensive care unit. Neurosurg Clin N Am. (2018) 29:299–314. 10.1016/j.nec.2017.11.008 29502719

[25] Arena F Vannetti F Di Pilato V Fabbri L Colavecchio OL Giani T. Diversity of the epidemiology of carbapenemase-producing Enterobacteriaceae in long-term acute care rehabilitation settings from an area of hyperendemicity, and evaluation of an intervention bundle. J Hosp Infect. (2018) 100:29–34. 10.1016/j.jhin.2018.05.025 29879446

[26] Mylotte JM Graham R Kahler L Young BL Goodnough S. Impact of nosocomial infection on length of stay and functional improvement among patients admitted to an acute rehabilitation unit. Infect Control Hosp Epidemiol. (2001) 22:83–7. 10.1086/501868 11232883

[27] Popović N Stefanović-Budimkić M Mitrović N Urošević A Milošević B Pelemiš M. The frequency of poststroke infections and their impact on early stroke outcome. J Stroke Cerebrovasc Dis. (2013) 22:424–9. 10.1016/j.jstrokecerebrovasdis.2013.03.003 23540255

[28] Suda S Aoki J Shimoyama T Suzuki K Sakamoto Y Katano T. Stroke-associated infection independently predicts 3-month poor functional outcome and mortality. J Neurol. (2018) 265:370–5. 10.1007/s00415-017-8714-6 29249057

[29] Rollnik JD. Outcome of MRSA carriers in neurological early rehabilitation. BMC Neurol. (2014) 14:34. 10.1186/1471-2377-14-34 24555811 PMC3932788

[30] Rollnik JD Bertram M Bucka C Hartwich M Jöbges M Ketter G. Outcome of neurological early rehabilitation patients carrying multi-drug resistant bacteria: results from a German multi-center study. BMC Neurol. (2017) 17:53. 10.1186/s12883-017-0833-2 28320357 PMC5359920

[31] Teasell R Bayona N Marshall S Cullen N Bayley M Chundamala J. A systematic review of rehabilitation of moderate to severe acquired brain injuries. Brain Inj. (2007) 21:107–12. 10.1080/02699050701201524 17364527

[32] Andriessen TM Horn J Franschman G van der Naalt J Haitsma I Jacob B. Epidemiology, severity classification, and outcome of moderate and severe traumatic brain injury: a prospective multicenter study. J Neurotrauma. (2011) 28:2019–31. 10.1089/neu.2011.2034 21787177

[33] Avesani R Roncari L Khansefid M Formisano R Boldrini P Zampolini M. The Italian National Registry of severe acquired brain injury: epidemiological, clinical and functional data of 1469 patients. Eur J Phys Rehabil Med. (2013) 49:611–8. 23558700

[34] Dani A. Colonization and infection. Cent European J Urol. (2014) 67:86–7. 10.5173/ceju.2014.01.art19 24982790 PMC4074726

[35] Banach DB Bearman G Barnden M Hanrahan JA Leekha S Morgan DJ. Duration of contact precautions for acute-care settings. Infect Control Hosp Epidemiol. (2018) 39:127–44. 10.1017/ice.2017.245 29321078

[36] Teasdale G Jennett B. Assessment of coma and impaired consciousness. A practical scale. Lancet. (1974) 2:81–4. 10.1016/S0140-6736(74)91639-0 4136544

[37] Hagen C Malkmus D Durham P “Levels of cognitive functions”. In: Rehabilitation of the Head-Injured Adult: Comprehensive Physical Management. Downey, CA: Professional Staff Association, Rancho Los Amigos Hospital (1997).

[38] Rappaport M Hall KM Hopkins K Belleza T Cope DN. Disability rating scale for severe head trauma: coma to community. Arch Phys Med Rehabil. (1982) 63:118–23. 10.1037/t29015-000 7073452

[39] Shah S Vanclay F Cooper B. Improving the sensitivity of the barthel index for stroke rehabilitation. J Clin Epidemiol. (1989) 42:703–9. 10.1016/0895-4356(89)90065-6 2760661

[40] Popovich KJ Hota B Hayes R Weinstein RA Hayden MK. Effectiveness of routine patient cleansing with chlorhexidine gluconate for infection prevention in the medical intensive care unit. Infect Control Hosp Epidemiol. (2009) 30:959–63. 10.1086/605925 19712033

[41] Borg MA Suda D Scicluna E. Time-series analysis of the impact of bed occupancy rates on the incidence of methicillin-resistant *Staphylococcus aureus* infection in overcrowded general wards. Infect Control Hosp Epidemiol. (2008) 29:496–502. 10.1086/588157 18510458

[42] Zheng Y He L Asiamah TK Otto M. Colonization of medical devices by staphylococci. Environ Microbiol. (2018) 20:3141–53. 10.1111/1462-2920.14129 29633455 PMC6162163

[43] Rosenthal VD Bat-Erdene I Gupta D Belkebir S Rajhans P Myatra SN. (2020). International nosocomial infection control consortium. International nosocomial infection control consortium (INICC) report, data summary of 45 countries for 2012-2017: device-associated module. Am J Infect Control. 48:423–32. 10.1016/j.ajic.2019.08.023 31676155

[44] Abraham P Lamba N Acosta M Gholmie J Dawood HY Vestal M. Antibacterial prophylaxis for gram-positive and gram-negative infections in cranial surgery: a meta-analysis. J Clin Neurosci. (2017) 45:24–32. 10.1016/j.jocn.2017.07.039 28802796

[45] Cao Y Pu K Li G Yan X Ma Y Xue K. The role of antibiotic prophylaxis in clean neurosurgery. World Neurosurg. (2017) 100:305–10. 10.1016/j.wneu.2016.12.108 28104524

[46] Doherty A McNicholas S Burger H Boldrini P Delargy M. European survey of management of patients with multidrug-resistant organisms in rehabilitation facilities. Eur J Phys Rehabil Med. (2019) 55:418–23. 10.23736/S1973-9087.19.05570-9 30781935

[47] Tarzi S Kennedy P Stone S Evans M. Methicillin-resistant *Staphylococcus aureus*: psychological impact of hospitalization and isolation in an older adult population. J Hosp Infect. (2001) 49:250–4. 10.1053/jhin.2001.1098 11740872

[48] Pike JH McLean D. Ethical concerns in isolating patients with methicillin-resistant *Staphylococcus aureus* on the rehabilitation ward: a case report. Arch Phys Med Rehabil. (2002) 83:1028–30. 10.1053/apmr.2002.33108 12098167

[49] World Health Organization Antimicrobial Resistance: Global Report on Surveillance. (2014). Available online at: https://apps.who.int/iris/handle/10665/112642 (accessed May 06, 2020).

